# New Sight for Old: Commentary On the Use of Pilocarpine for Presbyopia

**DOI:** 10.18502/jovr.v19i4.17786

**Published:** 2024-12-31

**Authors:** Cameron F. Parsa

**Affiliations:** ^1^Erasmus Hospital, Université Libre de Bruxelles, Brussels, Belgium; ^2^Faculty of Medicine, Sorbonne University, Paris, France; ^4^Cameron F. Parsa: http://orcid.org/0000-0001-6110-9294

It is always of interest to hear about potential new uses for established drugs. Pilocarpine was first isolated in 1874 and quickly became a mainstay for glaucoma treatment. Despite its understood and occasionally serious adverse effects with chronic use – established over the course of a century – the risk–benefit ratio firmly favored its utility in the treatment of otherwise irrevocable, blinding glaucoma.

However, over the last 50 years, as alternative topical drugs with greater efficacy and better safety profiles were introduced, the use of pilocarpine gradually decreased. It became essentially relegated to a niche category for acute use only, such as in the treatment of angle closure glaucoma, or as an aid in the diagnosis of Adie's pupil.

A few publications in recent years^[1,2]^ have studied or discussed the renewed chronic use of pilocarpine as a treatment option for presbyopia, a condition far more prevalent than glaucoma. Based on two randomized trials comprising 354 patients receiving 1.25% pilocarpine, followed for 30 days,^[3,4]^ United States FDA approval was granted in 2021 for such use.

Since this approval, ongoing studies,^[5]^ including one now by Mousavi and colleagues^[6]^ comprising 75 patients, have been undertaken to determine the efficacy of pilocarpine in presbyopia. The report by Mousavi and associates is the first in which pilocarpine was studied monocularly for this purpose, using the contralateral eye as a control.^[6]^ A comparison was also made between two distinct pilocarpine solutions to determine their relative efficacy. The authors found that the drops appeared safe in the small sample studied and noted a slightly “higher amplitude of accommodation and pupil constriction” with a more recently produced solution as compared to a prior commercially available one. They conclude their study by suggesting larger trials, including bilateral drop instillation, to further verify their findings and investigate potential safety issues.^[6]^

Such studies^[1][3,4][5][6]^ encompassing a relatively small number of patients, most with follow-up duration of only several weeks, did not report serious adverse effects. One wonders whether these recent studies were sufficiently powered to detect potentially serious or long-term side effects known to be associated with this drug. Indeed, since the 2021 FDA approval for presbyopia management, reports have emerged describing previously recognized potential secondary effects, such as retinal detachments.^[7,8]^

Some of this oversight could be attributed to the fact that older publications detailing such side effects are less accessible and have been neglected or forgotten. Despite its importance to medical researchers, PubMed still does not offer a comprehensive listing of papers published before 1965, with many of those now listed comprising only a title without an abstract or other content. A similar scenario involving lack of awareness of earlier publications led to the well-publicized and unfortunate death of a research study volunteer in 2001 at the Johns Hopkins Hospital. The study tested the effects of methacholine, an acetylcholine agonist that induces bronchoconstriction, in combination with hexamethonium bromide.^[9,10,11,12]^ The latter drug, a former antihypertensive drug used in the 1940s and 50s had been withdrawn from clinical use by the 1970s as superior antihypertensives became available.^[9,11,12]^ Unbeknownst to the investigators who employed standard search methods before obtaining Institutional Review Board (IRB) approval, hexamethonium bromide had already (between 1953 and 1962) been reported to occasionally cause potentially lethal pulmonary adverse effects.

Administrative reactions to this tragedy led to stricter IRB approval requirements for all research, including simple retrospective chart reviews. This had the unfortunate effect of slowing down the pace of medical research^[13]^ while doing little to address the core issue of the constrained accessibility of older published reports. Despite some pre-1965 citations being gradually incorporated into PubMed, it remains nevertheless inadequate for reviewing the literature included within the Index Medicus, published since 1879.

Interestingly, a perusal through the indices volume of the 1958–76 second edition of the encyclopedic *Duke-Elder System of Ophthalmology*, using the word “pilocarpine,” quickly revealed a wealth of information with early citations on the chronic topical use of pilocarpine and adverse effects.^[14]^

The adverse effects of prolonged pharmaceutical-induced pupillary miosis include iris cysts, often reversible, as first described by Vogt,^[15,16]^ and later referred to by Berliner as “pilocarpine cysts.”^[17]^ Posterior synechiae could also be noted, yet it was discovered that periodic dilation or concurrent vasoconstrictor administration^[18]^ could prevent their formation. In the prescribed use of pilocarpine for presbyopia (instilled once or twice daily rather than every six hours), some pupillary movement is expected, preventing such problems. Nevertheless, as some patients may be anticipated to use the drops more frequently, such effects may yet occur.

Prolonged iris stretch from chronic pilocarpine use is also known to produce atrophic and degenerative changes in the iris, and as many anterior segment surgeons have noticed, pupils that become difficult to dilate.

As mentioned earlier, the forward movement of the iris-lens diaphragm, along with the vitreous base, is known to help precipitate retinal detachments in susceptible patients.^[7,8]^ Despite warnings in the prescribing information, it is unlikely that many patients will undergo a full peripheral retinal examination prior to pilocarpine administration.^[8]^ Vitreofoveal traction can also occur.^[19]^ Today, OCT scanning of the disc and macula could be incorporated in ongoing pilocarpine trials. This may provide further evidence for Tolentino and Schepen's explanation that cystoid macular and disc “edema,” otherwise known as Irvine–Gass syndrome,^[20,21]^ results from traction caused by anterior vitreous displacement.^[22]^

Various forms of cataracts, beginning as vacuolar changes in and beneath the anterior capsule epithelium leading to potentially reversible iridescent opacities have also been noted with chronic four-times daily pilocarpine use.^[14,23]^ Continued drug use led to the development of persistent posterior cortical and nuclear changes. Such changes were restricted to the treated eye,^[14]^ and progression was no longer noted once the drug use ceased.^[14]^

The mechanism for such cataract formation can now be better understood. Forces applied at the interface of softer and harder lens layers can produce cortical wedge-shaped, or cuneiform, “shear stress” cataracts.^[24,25,26,27,28]^

The intense ciliary muscle spasms that occur intermittently with once or twice daily pilocarpine use for presbyopia could produce more frequent larger amplitude dynamic mechanical shear stress forces between outer cortical and nuclear crystalline layers to cause fiber disorganization or compaction. Detailed long-term follow-up of lenticular changes in such patients, including those treated monocularly, could lead to a better understanding of details of accommodative forces applied to the lens^[29]^ and, more notably, the development of common cortical cataracts.

Knowledge gained from such studies could lead to recommendations for earlier and stronger presbyopic spectacle correction. Once the crystalline lens becomes differentially rigid due to age, reducing ciliary muscle forces applied to effectuate changes in lens morphology might be a means to reduce the otherwise common and often stepwise development of cortical cataracts that typically begin with presbyopia.

It is also useful to review the physiological basis for purported gains in near vision. One wonders how the potential benefits of pilocarpine have been overlooked following its use. The overlap between patients treated with pilocarpine for glaucoma and those suffering from presbyopia has always been present. Yet after pilocarpine was replaced by other drugs there is no mention of glaucoma patients having asked to continue being treated with pilocarpine to counter their presbyopia.

Responding to that question and resolving any controversy relies on clearly defining what is meant by “accommodation.” As should be well-understood, accommodation refers to a dynamic process by which the eyes focus on objects at different distances and maintain clear imagery by varying the shape of the crystalline lens. Topical pilocarpine does *not* promote accommodation in this sense at all. By stimulating the parasympathetic system locally at the level of the ciliary muscle, it instead provokes a prolonged ciliary muscle *spasm, *or contraction, that is essentially static.^[2]^ The distinction in terminology is critical. Such continuous ciliary muscle contraction does not allow for dynamic focusing the eye at different distances – the very definition of accommodation – but merely holds the eye in a steady state near focus. The fact that true accommodation, a dynamic process, is not enhanced is easily verifiable in the clinic using dynamic retinoscopy or, if available, an abberometer.^[30,31,32]^ Imprecise use of language- interchanging accommodation,^[3,4]^ a dynamic action, with continuous ciliary muscle contraction or spasm^[2]^ – has led to a great deal of confusion.^[33]^

So, how have so many investigators reported improved near, and occasionally, distance vision? That can be partly attributed to another longstanding lack of precision in medical terminology. Authors investigating pilocarpine use in presbyopia have relied on the Donders method as a purported means to assess accommodative amplitude. However, the Donders method, where an object is brought closer to the subject until its image is blurred depends on both accommodation as well as pupillary size.^[34]^ The primary effect of pilocarpine is of promoting miosis, creating a pinhole effect that increases depth of field. Interestingly, in 1864, Donders himself correctly commented how the use of physostigmine, a prior discovered miotic agent, improved ametropia by decreasing pupil size rather than enhancing accommodation.^[35]^

In patients with early presbyopia, pharmaceutically induced ciliary muscle contractionwill produce a persistent near focus, facilitating near vision alongside reduced accommodative *effort *(a technique once used to reduce accommodative-driven convergence in children with esotropia). Despite the persistent lenticular focus for near vision, distance vision may not be appreciably degraded due to the pinhole effect induced by miosis, which increases depth of field. This, however, occurs at the expense of optimal light exposure, the very purpose of a variable-sized pupil.

In older individuals with an essentially rigid crystalline lens, pharmaceutical spasm of the ciliary muscle will not significantly affect lens morphology, and any gains in near vision will be obtained solely through the pinhole effect. However, a similar effect could be achieved using artificial pupils integrated into contact lenses.^[36]^ Alternatively, miosis could be simply induced via the pupillary light reflex following brighter illumination.

As the crystalline lens becomes increasingly rigid over time and accommodative effort intensifies, physiologic recruitment of miosis via the near reflex triad also increases.^[37]^ With persistent iris stretch, older individuals thus tend to develop smaller pupils (“senile miosis”). Such miosis is partially released during distance viewing, allowing for improved retinal illuminance. For older individuals whose retinal sensitivity to light may already be diminished with age, bilateral use of pilocarpine drops, whose effects persist into the evening, will inevitably reduce retinal illuminance, making night driving more hazardous, despite some patients stating subjective responses to the contrary.^[1,38]^ Indeed, inferior driving skills were objectively noted following pilocarpine administration.^[38]^ Mousavi and coworkers investigated using pilocarpine in one eye alone.^[6]^ While this approach was intended to allow the contralateral eye to serve as a control, it also helped mitigate some of the aforementioned issues. However, the resulting unequal pupil sizes (anisocoria) could be unsightly for some patients, particularly those with lightly colored irides, as the authors point out.^[6]^ Another concern to keep in mind is the induction of Pulfrich phenomenon.^[2]^ Due to the inter-eye discrepancies in illumination, dynamic stereoscopic perception will be affected, posing difficulties in distance estimation for activities such as driving, or during sport activities, with illusory three-dimensional pathways created during motion. Reports have revealed accidents occurring as a result of unilateral induced miosis, while patients suffering from Pulfrich phenomenon due to other causes (i.e., optic neuritis) have reported difficulties with driving.^[39]^

The variable contralateral pupil size throughout the day coupled with the intermittency of pilocarpine treatment does not allow for adaptation to the Pulfrich phenomenon.^[40]^ Proposed solutions, such as balancing retinal illumination with a neutral density filter over the contralateral eye^[40,41]^ would create the same issues caused by bilateral dim retinal illumination, as discussed earlier.^[38]^

In light of facts regarding potential pathological side effects mentioned earlier and the lack of study power or long-term follow-up in recent studies leading to FDA approval,^[3,4]^ one might reconsider the risks of using pilocarpine in patients with presbyopia. The wisdom of its renewed chronic use for a non-blinding condition appears difficult to justify, especially when time-tested, more effective and safer alternatives exist, such as spectacle correction for near focus and adequate lighting to induce adequate miosis.

As in the tale of Aladdin where new lamps were offered for old, or perhaps in this case, new sight for old, experience teaches us to maintain a healthy reserve.
